# Punicalagin attenuates ventricular remodeling after acute myocardial infarction via regulating the NLRP3/caspase-1 pathway

**DOI:** 10.1080/13880209.2023.2224403

**Published:** 2023-06-25

**Authors:** Jian-fei Peng, Xiao-ni Zhao, Meng Zhang, Jing-ya Li, Chun-chun Zhao, Shu-shu Wang, Jia-li Wang, Hui Shi, Peng Zhou, Liang Wang

**Affiliations:** a Department of Integrated Traditional Chinese and Western Medicine, Anhui University of Chinese Medicine, Hefei, Anhui, China; b Research Institute of Integrated Traditional Chinese and Western Medicine, Anhui Academy of Chinese Medicine, Hefei, Anhui, China

**Keywords:** Natural products, molecular docking, inflammatory response, cardiovascular disease

## Abstract

**Context:**

Punicalagin has myocardial protection; the mechanism of punicalagin on ventricular remodeling (VR) after acute myocardial infarction (AMI) remains unclear.

**Objective:**

These studies explore the role and mechanism of punicalagin in preventing and treating VR after AMI.

**Materials and methods:**

Molecular docking was used to predict the targets of punicalagin. After 2 weeks of AMI model, the SD rats were randomly divided into model, and punicalagin (200, 400 mg/kg, gavage) groups for 4 weeks. Thoracotomy with perforation but no ligature was performed on rats in control group. The protein expression of nucleotide-binding oligomerization domain-like receptor family pyrin domain-containing 3 (NLRP3), apoptosis speck-like protein (ASC), caspase-1, gasdermin D (GSDMD), and GSDMD-N, the mRNA expression of NLRP3, caspase-1, GSDMD, interleukin-1β (IL-1β) and IL-18 were evaluated.

**Results:**

Punicalagin had binding activities with NLRP3 (Vina score, −5.8), caspase-1 (Vina score, −6.7), and GSDMD (Vina score, −6.7). Punicalagin could improve cardiac function, alleviate cardiac pathological changes, minimize the excessive accumulation of collagen in the left ventricular myocardium (*p* < 0.01), and inhibit cardiomyocyte apoptosis (*p* < 0.01). Furthermore, punicalagin could inhibit the overexpression of NLRP3, caspase-1, and GSDMD via immunohistochemistry (*p* < 0.01). Punicalagin inhibited the protein levels of NLRP3, caspase-1, ASC, GSDMD, and GSDMD-N (*p* < 0.05, *p* < 0.01). Punicalagin reduced the mRNA expression of NLRP3, caspase-1, GSDMD, IL-1β and IL-18 (*p* < 0.05, *p* < 0.01).

**Conclusions:**

Punicalagin may provide a useful treatment for the future myocardial protection.

## Introduction

Ischemic heart disease (IHD) is the main cause of death and morbidity worldwide. Acute myocardial infarction (AMI) is the leading cause of death in patients with IHD (Shen et al. 2022). The most important predictors of ventricular remodeling (VR) are the site and severity of the AMI. VR is a long-term pathological change that leads to heart failure (HF), which is a major cause of death in patients with AMI. Vessel endothelial damage and extracellular matrix deposition, as well as apoptosis and necrosis of cardiomyocytes, are among the most significant alterations in cardiac microstructures that occur with the progression of VR (Sokolova et al. 2019). Numerous clinical and experimental studies have established that inflammation plays a critical role in VR, and the nucleotide-binding oligomerization domain-like receptor family pyrin domain-containing 3 (NLRP3) inflammasome has garnered widespread attention as a central inflammatory response (Yan et al. 2021). Pyroptosis has been shown to be crucial in the process of acute reperfusion and subsequent VR (Shen et al. 2021). The NLRP3 inflammasome is a multiprotein complex that interacts with caspase-1 to promote the maturation and release of pro-inflammatory chemicals, such as interleukin-1β (IL-1β) and IL-18, which is found in the inflammatory response, therefore triggering pyroptosis (Lei et al. 2018). As a result, investigations have demonstrated that the NLRP3 inflammasome is critical in the progression of VR. Treatment of MI/R damage and VR with drugs that inhibit NLRP3-mediated pyroptosis may be an important therapeutic target, and it has a major influence on the development of and prognosis for AMI.

Increased consumption of fruits and nuts is extremely beneficial to human health and can help to prevent several diseases. In terms of health nutrition and medicine, there is a growing interest in the study of food composition at present (Venusova et al. 2021). Punicalagin is the primary ellagitannin polyphenol found in the peel or seeds of the pomegranate, raspberries, strawberries, and walnuts (Hering et al. 2021), and it possesses anticancer, antiinflammatory, antioxidant, and antiviral properties (Xu et al. 2021). Punicalagin is effective in the treatment of a variety of diseases, including cervical cancer (Xie et al. 2022), lung cancer (Fang et al. 2021), acute leukemia (Subkorn et al. 2021), lupus nephritis (Seo et al. 2020), diabetes and its complications (Hua, Han, et al. 2022; Liu et al. 2022) bronchial asthma (Yu and Li 2022), rheumatoid arthritis (Ge et al. 2022), Alzheimer’s disease (Xu et al. 2022), and acute myocardial infarction (Ding et al. 2017). Punicalagin has good myocardial protection, which significantly enhanced cardiac function, decreased the size of infarcts, decreased CK-MB and LDH activity, and inhibited cardiomyocyte apoptosis (Ding et al. 2017). These studies indicated that punicalagin has good biological activity on AMI and is worthy of further study. Additionally, by suppressing the expression of NLRP3 and caspase-1, punicalagin was able to alleviate cell death caused by inflammation, as shown by the reduction of inflammatory cell death mediated by the production of IL-1β and IL-18 (An et al. 2020). However, it remains unclear whether the protective mechanism of punicalagin on VR after AMI is related to the regulation of NLRP3/caspase-1 signaling pathway.

The study examined the effect of punicalagin on the NLRP3/caspase-1 signaling pathway. Molecular docking was used to predict the relationship between punicalagin and NLRP3, and to explore the role of punicalagin *in vivo* underlying molecular mechanisms in the treatment of AMI.

## Materials and methods

### Animals

Sprague-Dawley (SD) rats (male, weight = 200 ± 20 g) were acquired from Anhui Medical University’s Experimental Animal Center (SCXK: 2017-001). All experiments adhered to the guidelines established by Anhui University of Chinese Medicine’s institutional animal care and use committee (AHUCM-rats-2021077).

### Chemicals and materials

Punicalagin, the purity is 98%, purchased from Herbpurify Co., Ltd. (Chengdu, China). Anti-NLRP3, Anti-GSDMD, and Anti-GAPDH were purchased from Abcam (Cambridge, MA, USA). Anti-cleaved-caspase 1 and anti-ASC were purchased from Affinity Biosciences (Cincinnati, OH, USA).

### Molecular docking of punicalagin and the targets

CB-dock docking platform was used to predict the degree of binding between punicalagin and the targets (NLRP3, caspase-1 and GSDMD). The chemical structure of punicalagin was downloaded from the PubChem website, and PDB formats of NLRP3 (ID: 6npy) (Abdullaha et al. 2020), caspase-1 (ID: 1rwx) (Patel et al. 2021), and GSDMD (ID: 5wqt) (Kuang et al. 2017) were downloaded from RCSB (https://www.rcsb.org/). The SDF file of punicalagin was uploaded to ‘the ligand’, and the PDB file of the target was uploaded to ‘the protein’, the submit docking (Liu et al. 2020).

### Establishment of AMI rat model

As previously mentioned, the AMI rat model was established by ligation of the left anterior descending artery (LAD) coronary artery (Wang et al. 2020). Briefly, rats were anesthetized with isoflurane and equipped with a respirator to reduce the pain. The left thorax of the rat was opened and ligated 2 mm below the left atrial appendage with 6–0 silk thread. Thoracotomy with perforation but no ligature was performed on rats in control group.

### Drug treatment

After building the AMI model for 2 weeks, the rats were randomly divided into three groups (*n* = 10): model (i.g. deionized water), punicalagin (i.g. 200 mg/kg, dissolved in deionized water) and punicalagin (i.g. 400 mg/kg, dissolved in deionized water) for 4 weeks. Gavage was used to provide an identical volume of deionized water to the sham group.

### Hemodynamic indexes

Thirty minutes after the final gavage, the rats were anesthetized with pentobarbital sodium and a polystyrene catheter was inserted into the left ventricle via the right carotid artery. The PowerLab device was able to identify hemodynamic indications such as LVSP, LVEDP, +dp/dtmax, and − dp/dtmax.

### Myocardial histopathology

Left ventricular myocardium including myocardial infarction region was extracted, fixed with 4% paraformaldehyde, and sectioned into 5 μm thick paraffin-embedded tissue. Hematoxylin-eosin (HE) staining and Masson staining were used to observe the pathological changes, collagen deposition in the myocardial interstitium and collagen volume fraction (CVF).

### TUNEL staining

In each slice, five visual fields were randomly selected for microscopic examination, and the total number of cardiomyocytes and the apoptotic number of cardiomyocytes were counted in each field. The calculation formula is as follows: apoptosis rate = apoptotic cardiomyocytes number/all cardiomyocytes number × 100%.

### Immunohistochemical analyses

Primary antibodies were used to stain samples from the left ventricle, such as NLRP3, caspase-1, and GSDMD. They were incubated overnight at 4 °C, followed by secondary antibodies. Following that, DAB was used to stain the portions. Finally, pictures of the tissues were obtained using a fluorescent microscope.

### Western blotting for protein expression

For the determination of the total protein content in the myocardium, BCA kit was utilized. SDS-PAGE separates the same amount of protein and transfers it to the NC membrane, the cells were blocked in 5% skimmed milk powder for 2 h, before being incubated overnight at 4 °C. Anti-NLRP3 (1:1000), anti-caspase-1 p20 (1:1000), anti-GSDMD (1:1000), anti-GSDMD-N (1:1000), and anti-ASC (1:1000) antibodies were used in the study. In the following step, the primary antibody was incubated at 4 °C overnight before being incubated at room temperature with the secondary antibody for 2 h. After three washes with TBST, electrogenerated chemiluminescence (ECL) was employed to develop and fix the samples, followed by imaging using a gel imager. The experiment was carried out three times.

### RT-PCR for mRNA expression

The Trizol reagent was used to extract total RNA from myocardial of the left ventricular myocardium. Then the total RNA was reverse transcribed into cDNA. cDNA was synthesized from total cellular RNA using a RevertAid First Strand cDNA Synthesis Kit. RT-PCR was performed using LightCycler^®^ 96 PCR instrument (Roche, Switzerland). The results were analyzed Using 2^-ΔΔCq^ method to evaluate the mRNA levels of NLRP3, caspase-1, GSDMD, IL-1β and IL-18. The primers used were NLRP3 (Forward Sequence: 5′⟶3′, GAGCTGGACCTCAGTGACAATGC, Reverse Sequence: 5′⟶3′, AGAACCAATGCGAGATCCTGACAAC), caspase-1 (Forward Sequence: 5′⟶3′, GCACAAGACTTCTGACAGTACCTTCC, Reverse Sequence: 5′⟶3′, GCTTGGGCACTTCAATGTGTTCATC), GSDMD (Forward Sequence: 5′⟶3′, CAGCAGGCAGCATCCTTGAGTG, Reverse Sequence: 5′⟶3′, CCTCCAGAGCCTTAGTAGCCAGTAG), IL-1β (Forward Sequence: 5′⟶3′, AATCTCACAGCAGCATCTCGACAAG, Reverse Sequence: 5′⟶3′, TCCACGGGCAAGACATAGGTAGC), IL-18 (Forward Sequence: 5′⟶3′, CGACCGAACAGCCAACGAATCC, Reverse Sequence: 5′⟶3′, GTCACAGCCAGTCCTCTTACTTCAC).

### Statistical analysis

The SPSS 26.0 software was used to process all of the data, which was represented as mean ± standard deviation. Statistics were utilized to determine the difference between groups was statistically significant using a one-way ANOVA and the LSD test. *p*** **<** **0.05 indicates that the difference is statistically significant.

## Results

### Molecular docking results

**Figure 1.**
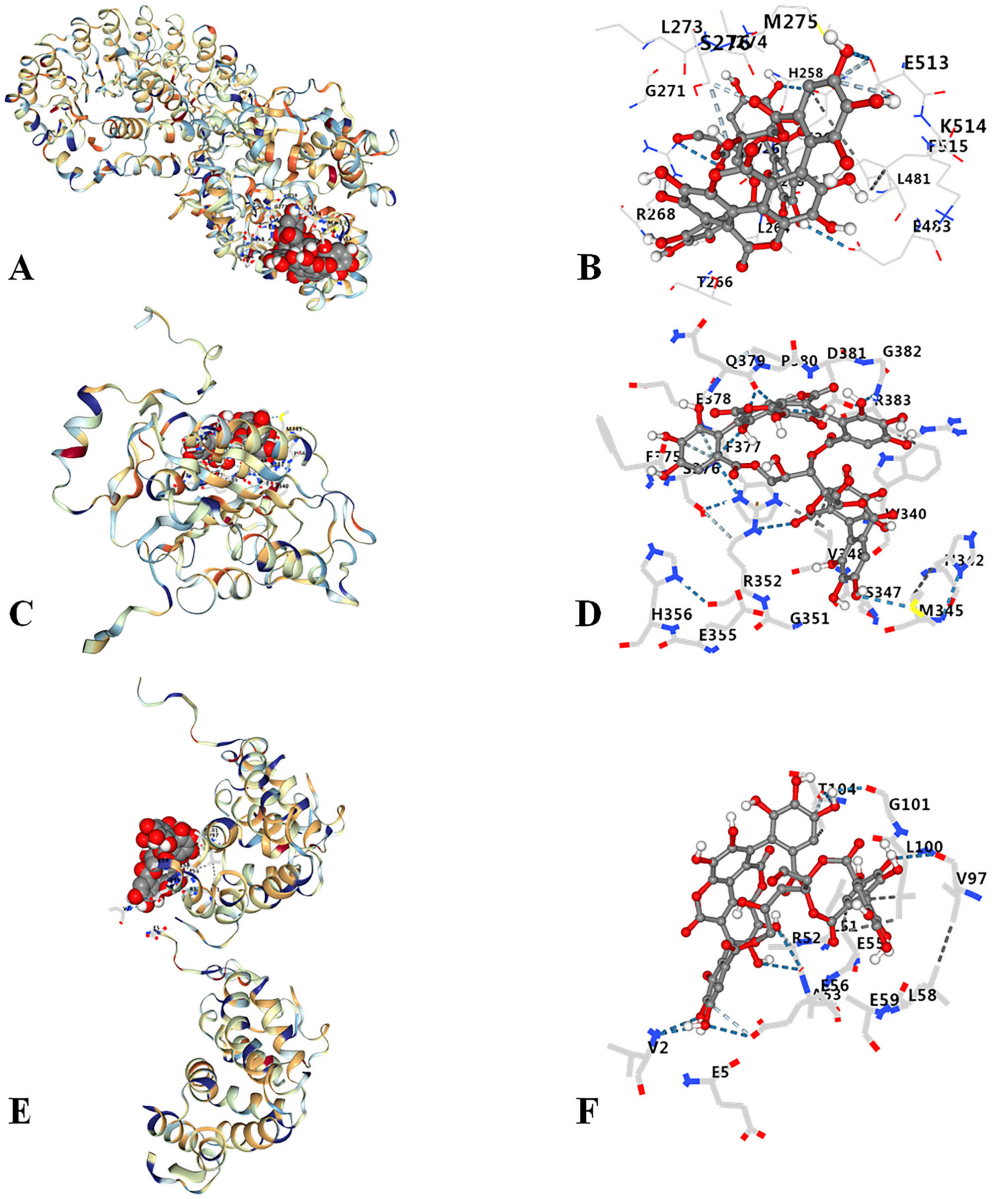
Molecular docking of punicalagin with NLRP3, caspase-1 and GSDMD. (A) 3D complex of punicalagin-NLRP3, (B) amino acid binding of punicalagin to NLRP3, (C) 3D complex of punicalagin-caspase-1, (D) amino acid binding of punicalagin to Caspase-1, (E) 3D complex of punicalagin-GSDMD, (F) amino acid binding of punicalagin to GSDMD.

Punicalagin had good binding activities with NLRP3 (Vina score, −5.8), caspase-1 (Vina score, −6.7), and GSDMD (Vina score, −6.7) (Table 1). 3D complex and amino acid binding of punicalagin to NLRP3, caspase-1 and GSDMD were showed in Figure 1. Results have indicated that punicalagin had a certain binding ability with relevant targets on NLRP3/Caspase-1/GSDMD signal pathway. The docking results were further verified by *in vivo* experiments.

**Table 1.** Docking results of punicalagin with NLRP3, caspase-1 and GSDMD.

Targets	Vina score	Cavity score	Center (x, y, z)	Size (x, y, z)
NLRP3	−5.8	12730	88, 94, 81	35, 34, 35
Caspase-1	−6.7	685	41, 65, 6	26, 26, 26
GSDMD	−6.7	868	30, −83, 41	26, 26, 26

### Punicalagin improved cardiac function following AMI

**Figure 2.**
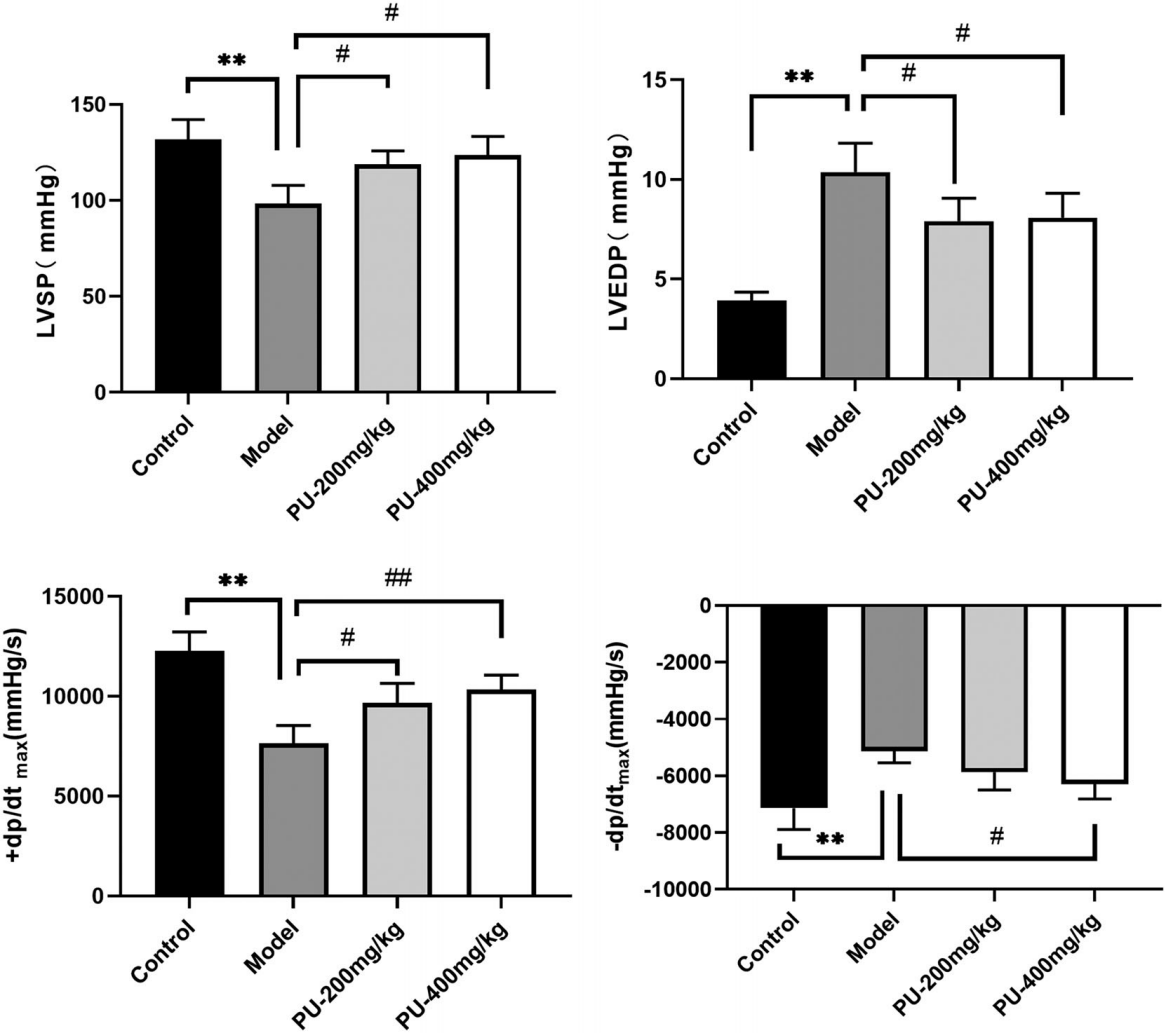
Punicalagin improved cardiac function on hemodynamic indexes. (A) LVSP; (B) LVEDP; (C) +dp/dtmax; (D) −dp/dtmax. The values are expressed as mean ± SD (*n* = 10). **p < 0.01 vs. control group; ^#^*p* < 0.05; ^##^*p* < 0.01 vs. model group.

Hemodynamic indexes reflect the size of the myocardial contraction or relaxation capacity. LVSP, +dp/dtmax, and − dp/dtmax were considerably lowered in the model group (*p*** **<** **0.01), whereas LVEDP was significantly raised when compared to the control group (*p*** **<** **0.01). With doses of 200 and 400 mg/kg, punicalagin lowered LVSP, +dp/dtmax, and − dp/dtmax and increased LVEDP, respectively (*p*** **<** **0.01, *p*** **<** **0.05) (Figure 2).

### Punicalagin improved the morphological changes via HE staining

**Figure 3.**
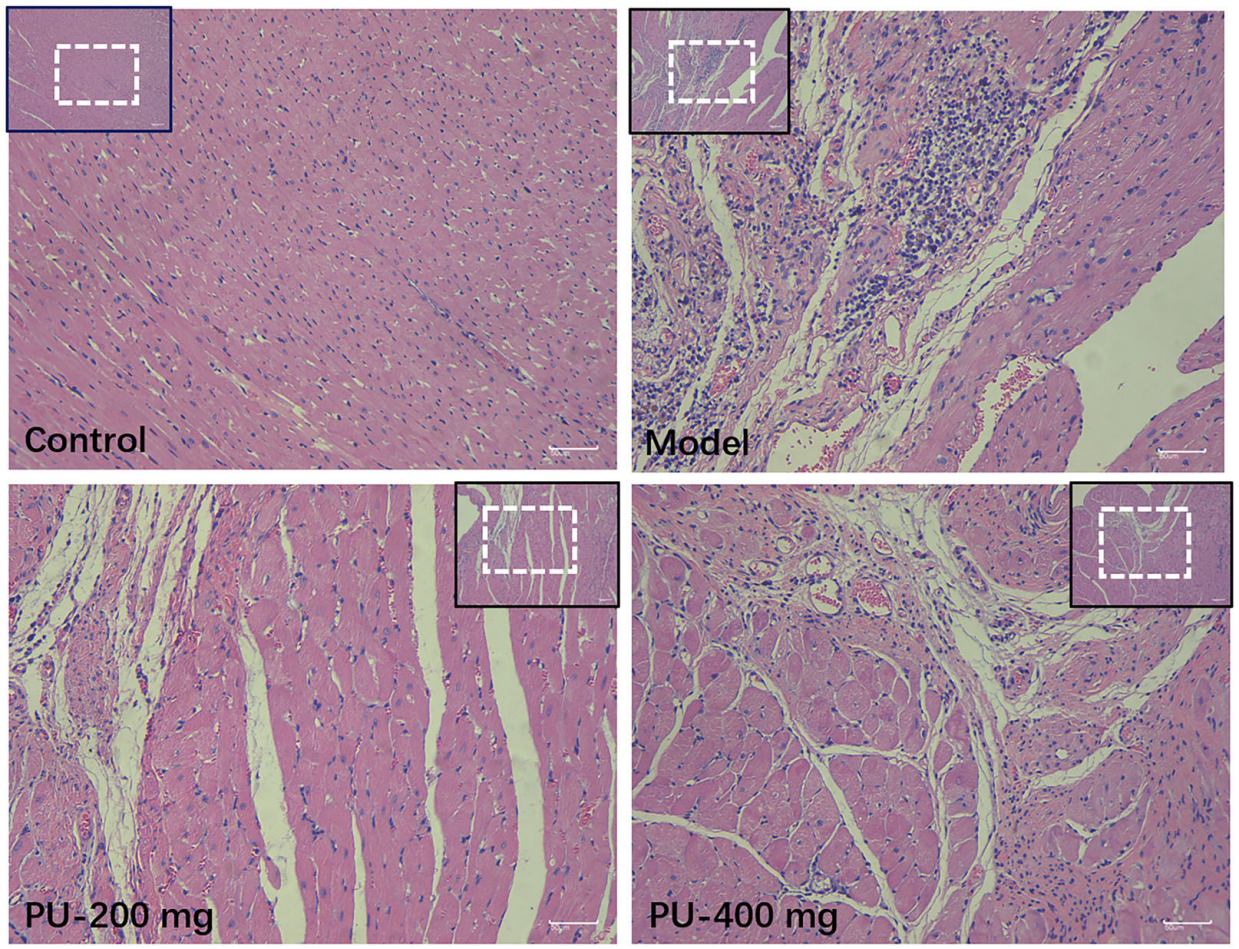
Punicalagin improved the morphological changes *via* HE staining (×100 and ×200).

Myocardial tissue in the control group had a normal morphology, with a smooth texture, an ordered arrangement of myocardial cells, and fewer cardiac fibroblasts. After punicalagin treatment, cardiomyocytes were arranged more regularly, fibroblasts were significantly reduced, and inflammatory cell infiltration was less. Punicalagin could alleviate cardiac pathological changes in AMI rats (Figure 3).

### Punicalagin alleviated myocardial fibrosis and CVF via Masson staining

**Figure 4.**
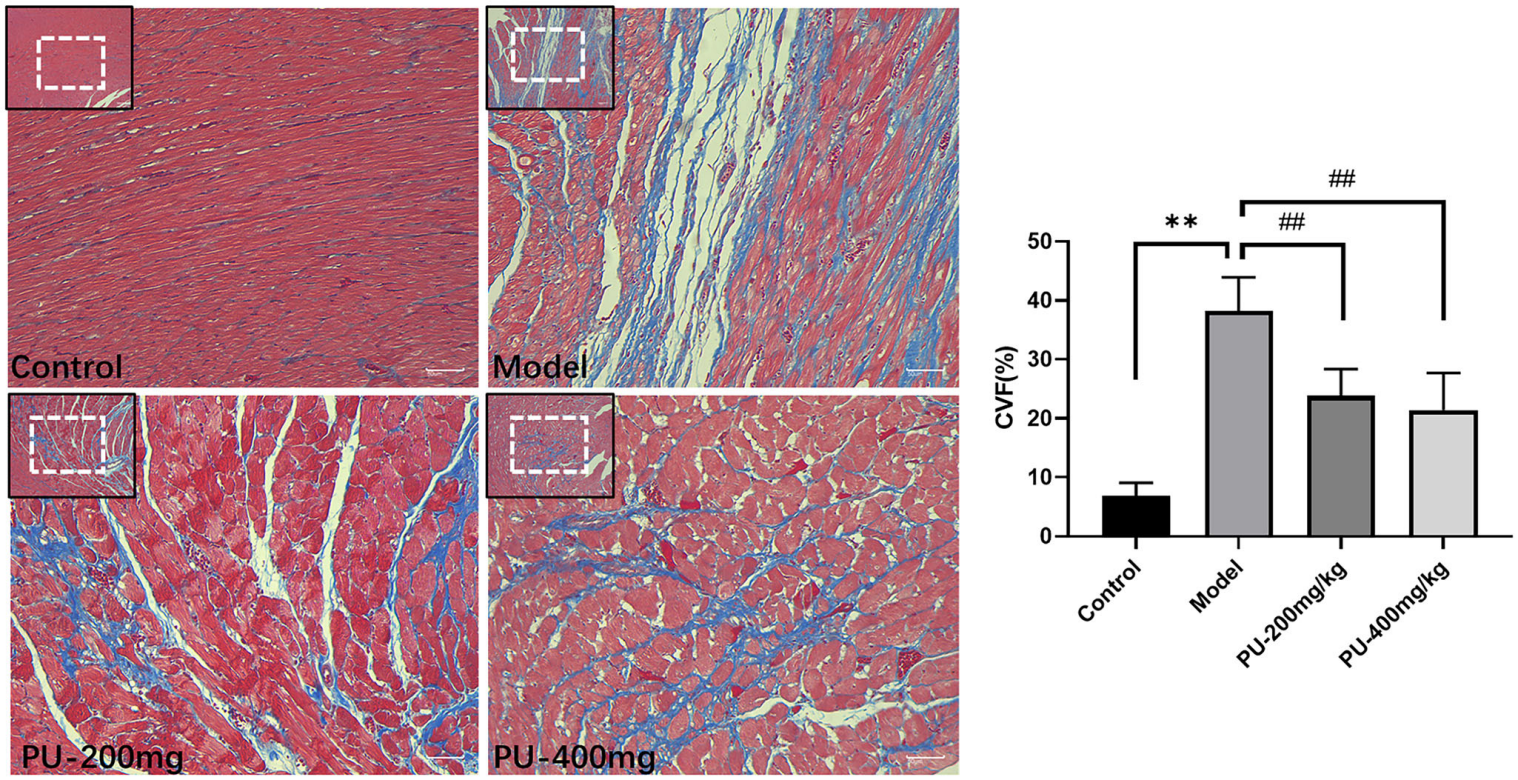
Punicalagin alleviated myocardial fibrosis and CVF via Masson staining (×100 and ×200). Values are expressed as mean ± SD (*n* = 10). **p < 0.01 vs. Sham group; ^##^*p* < 0.01 vs. model group.

Collagen volume fraction (CVF) can be used to observe the morphologic changes in the left ventricular myocardium. Control group rats had myocardium that was well-organized, had a limited number of collagen fibers, and had a uniform distribution of collagen fibers. In the model group, the myocardium was disorganized, collagen levels were elevated, the distribution was disorganized, and CVF levels were considerably higher (*p*** **<** **0.01). A considerable reduction in the collagen of cardiac tissue was seen after pretreatment with punicalagin. Additionally, the myocardial fibrosis was greatly alleviated, and the CVF was significantly lowered after pretreatment with punicalagin (*p*** **<** **0.01) (Figure 4).

### Punicalagin reduced cardiomyocyte apoptosis via TUNEL staining

**Figure 5.**
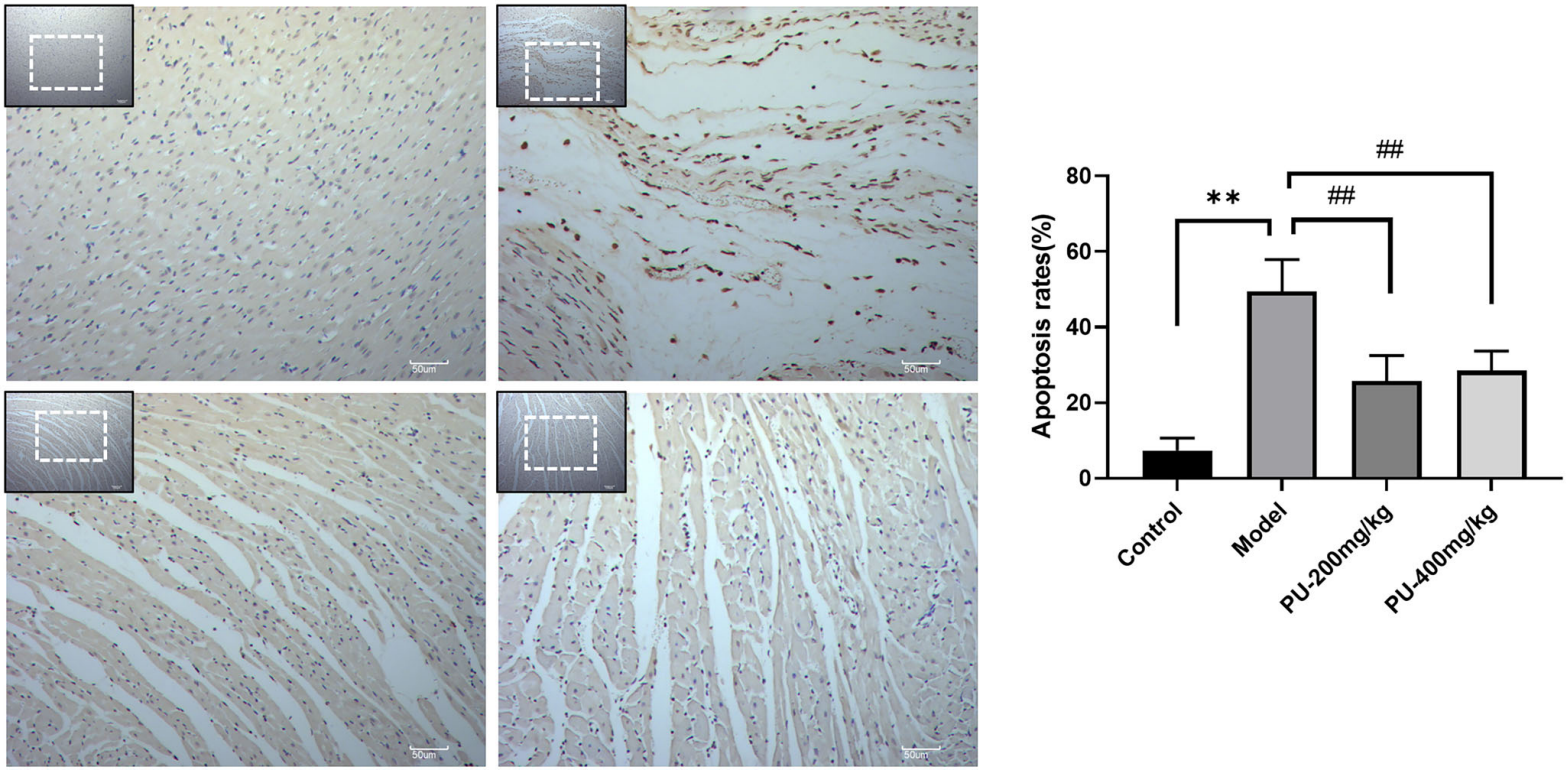
Punicalagin reduced cardiomyocyte apoptosis via TUNEL staining. The values are expressed as mean ± SD (*n* = 10). **p < 0.01 vs. Sham group; ^##^*p* < 0.01 vs. model group.

It was found that the model group’s nucleus was pale brown, and the apoptosis rate was substantially higher than that of the control group (*p*** **<** **0.01). Punicalagin therapy dramatically reduced the number of pale brown nuclei and apoptosis in comparison to the model group (*p*** **<** **0.01) (Figure 5).

### Punicalagin decreased NLRP3, caspase-1, and GSDMD levels via immunohistochemical analyses

**Figure 6.**
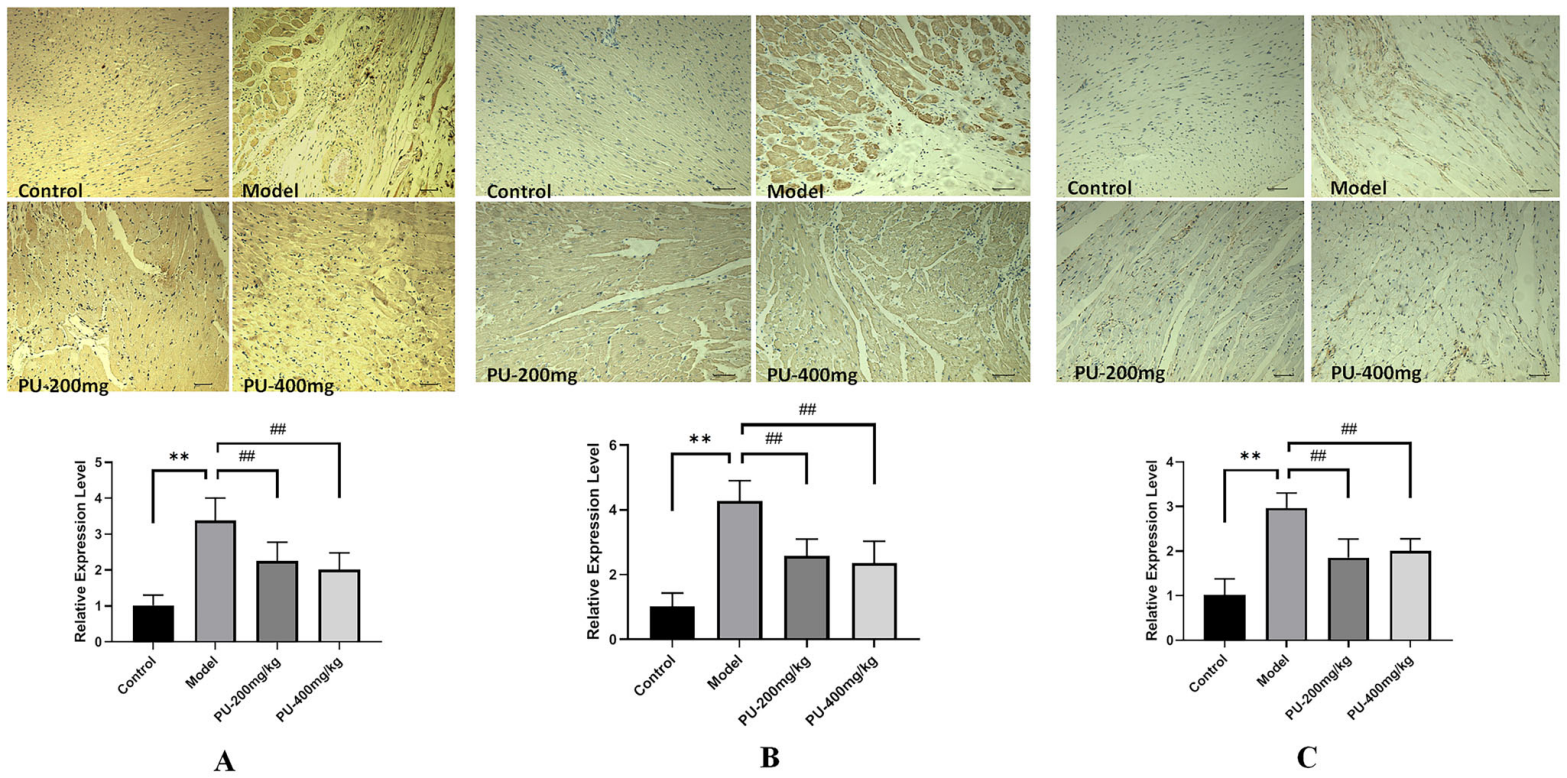
Punicalagin decreased NLRP3, caspase-1, and GSDMD levels via immunohistochemical analyses. (A) NLRP3; (B) caspase-1; (C) GSDMD. The values were expressed as the mean ± SD (*n* = 3); **p < 0.01 vs. control group; ^##^*p* < 0.01 vs. model group.

In comparison to the control group, the levels of NLRP3, caspase-1, and GSDMD were significantly increased in the model group (*p*** **<** **0.01). Compared with the model group, punicalagin decreased the expressions of NLRP3, caspase-1, and GSDMD (*p*** **<** **0.01) (Figure 6).

### LGZGD downregulated the protein expressions of NLRP3, caspase-1, ASC, GSDMD, and GSDMD-N

**Figure 7.**
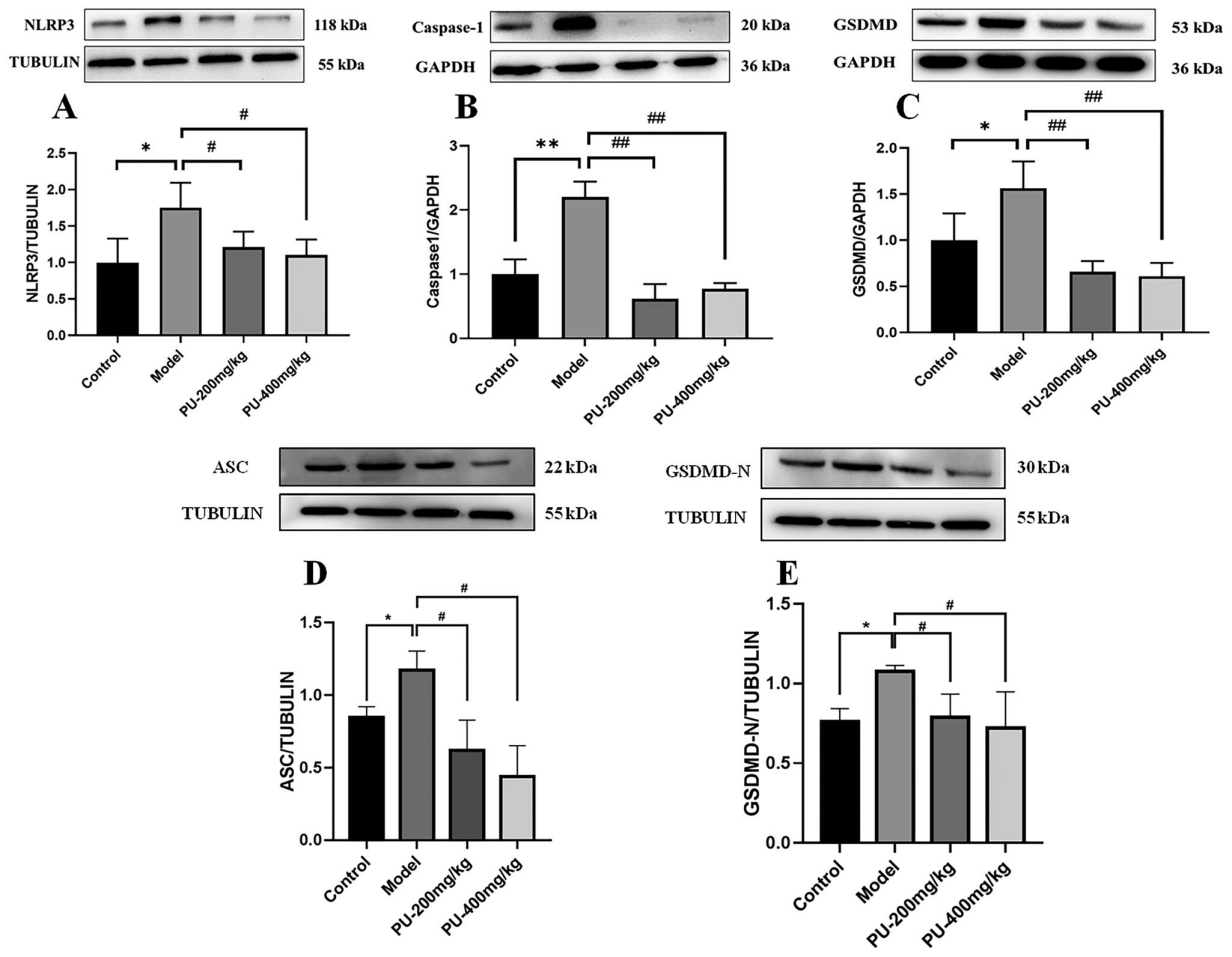
LGZGD downregulated the protein expression of NLRP3, caspase-1, GSDMD, ASC, and GSDMD-N. (A) NLRP3; (B) caspase-1; (C) GSDMD; (D) ASC; (E) GSDMD-N. The values were expressed as the mean ± SD (*n* = 3); *p < 0.05; **p < 0.01 vs. control group; ^#^*p* < 0.05; ^##^*p* < 0.01 vs. model group.

In comparison to the control group, the model group’s NLRP3, caspase-1, ASC, GSDMD, and GSDMD-N protein expression levels increased considerably (*p*** **<** **0.01, *p*** **<** **0.05). Compared with the model group, punicalagin could decrease the NLRP3, caspase-1, ASC, GSDMD, and GSDMD-N protein expression levels (*p*** **<** **0.01, *p*** **<** **0.05) (Figure 7).

### LGZGD inhibited the mRNA expression of NLRP3, caspase-1, GSDMD, IL-1β and IL-18

**Figure 8.**
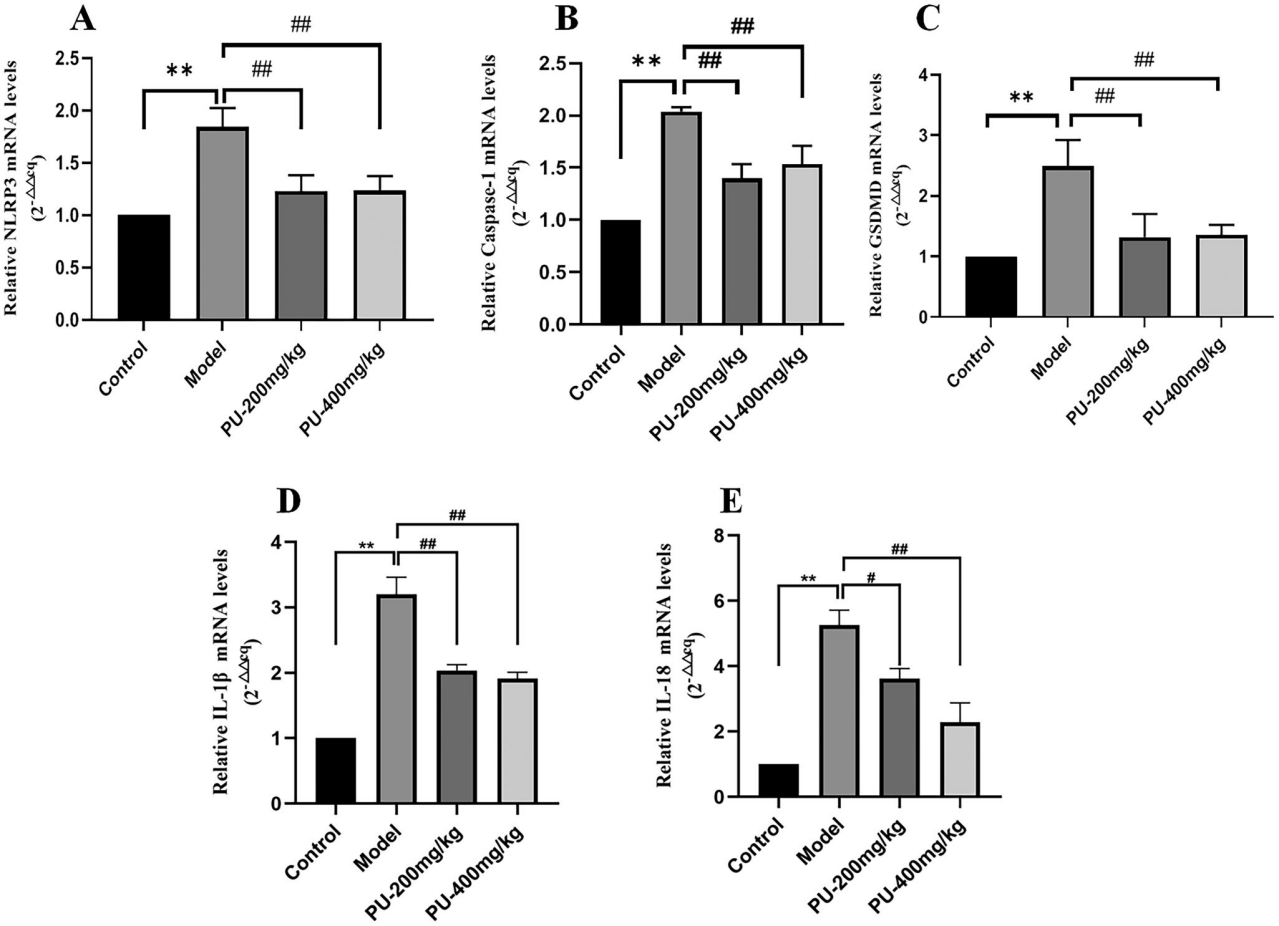
LGZGD inhibited the mRNA expression of NLRP3, caspase-1, GSDMD, IL-1β and IL-18. (A) NLRP3; (B) caspase-1; (C) GSDMD; (D) IL-1β; (E) IL-18. The values were expressed as the mean ± SD (*n* = 3); **p < 0.01 vs. control group; ^#^*p* < 0.05; ^##^*p* < 0.01 vs. model group.

Compared with the control group, the mRNA expression of NLRP3, caspase-1, GSDMD, IL-1β and IL-18 in the model group increased considerably (*p*** **<** **0.01). Punicalagin could reverse the results (*p*** **<** **0.01, *p*** **<** **0.05) (Figure 8).

## Discussion

VR is a gradual pathological alteration in the anatomy of the heart that can manifest itself as concentric remodeling, eccentric hypertrophy, or myocardial infarction (He et al. 2018). The creation of an NLRP3 inflammasome might eventually culminate in the release of excessive amounts of pro-inflammatory cytokines, triggering an inflammatory cascade. The NLRP3 inflammasome has been extensively studied and shown to have a significant role in the progression of VR following AMI, making it extremely necessary to investigate its mechanism of action. The activation of the NLRP3 inflammasome in injured cardiomyocytes is linked with cell death and inflammatory injury, both of which contribute to the progression of cardiac injury (Suetomi et al. 2018). The NLRP3 inflammasome is composed of NLRP3, apoptosis speck-like protein (ASC), and caspase-1. When NLRP3 is activated by DAMPs or PAMPs, it recruits the adaptor protein ASC linking NLRP3 and Pro-caspase-1, resulting in the formation of the NLRP3 inflammasome. Pro-caspase-1 is a proteolytic enzyme that automatically cleaves active caspase-1, which then activates IL-1β and IL-18, resulting in an excessive release of inflammatory cytokines and the induction of inflammatory programmed cell death (pyroptosis) (Buckley and Libby 2019; Silvis et al. 2021). Thus, pyroptosis mediated by the NLRP3 inflammasome is important in the process of VR after AMI. In order to alleviate AMI following VR, it is necessary to inhibit the activity of the NLRP3 inflammasome and reduce the overexpression of inflammatory factors.

Currently, chemical compounds with NLRP3 inflammasome inhibitory activity have been reported, mainly including glyburide, 16673-34-0, JC-124, MCC950, Bay 11-7082, OLT1177, INF4E, tranilast, CY-09, colchicine, and hydrogen sulfide. Most of the NLRP3 inhibitors by interfering with NLRP3-ASC connections, ATP-binding domain blockage with loss of ATPase function, blockade of NLRP3 posttranslational modification, Caspase-1 suppression, and NT-GSDMD pore formation (Mezzaroma et al. 2021). Some natural products have also been reported to have good NLRP3 inflammasome inhibitory activity. Phenolic compounds with NLRP3 inflammasome inhibitory activity are ellagic acid, curcumin, polydatin, phloretin, resveratrol, pterostilbene, salidroside, ferulic acid, gallic acid, and paeonol. Terpenoids with NLRP3 inflammasome inhibitory activity are oridonin, triptolide, artemisinin, celastrol, geniposide, betulin, ginsenoside Rg3, dioscin, and sweroside (Hua, Shi, et al. 2022). Quinone compounds with NLRP3 inhibitory activity are cryptotanshinone, tanshinone IIA, aloin, chrysophanol, rhein, emodin, aloe-emodin, shikonin, and plumbagin (Zhou et al. 2022). Foods or ingredients added to foods also have good myocardial protection (Lim et al. 2016). Punicalagin could significantly increase cell viability, inhibit LDH release, and suppress cell apoptosis, enhance the expression of nuclear Nrf2 and HO-1 in DOX-treated H9c2 cells, which alleviated the cardiotoxicity of DOX via Nrf2/HO-1 signaling pathway (Ye et al. 2019). Punicalagin also could enhance cardiac function, reduce myocardial infarction, decrease myocardial oxidative/nitrosative stress, reduce levels of IL-6, TNF-α, and inhibit IκB-α phosphorylation and NF-κB nuclear translocation, which decreased MI/R-induced inflammation by activating NRF-2-HO-1 signaling pathway (Yu et al. 2019). Inhibition of hyperglycemia-induced mitochondrial oxidative damage and cardiomyopathy by punicalagin through the regulation of the PTP1B-Stat3 pathway protected against diabetic cardiomyopathy (DCM) (Fu et al. 2021). Therefore, it is of great significance to study whether punicalagin reduces AMI after VR by inhibiting the NLRP3 signaling pathway, which can reveal the protective mechanism of punicalagin on the myocardium.

In this study, molecular docking prediction revealed that punicalagin may act on NLRP3. *In vivo* pharmacodynamics studies showed that punicalagin could improve hemodynamics and cardiac function, alleviate myocardial fibrosis and reduce myocardial cell apoptosis. Moreover, punicalagin could inhibit the expression of key proteins and genes in the NLRP3/Caspase-1 signaling pathway using immunohistochemistry, RT-PCR and Western blotting. The results suggested that punicalagin can prevent and treat VR after AMI, and the protective effect is related to its regulatory NLRP3/Caspase-1 signaling pathway. However, urolithin A, as a metabolite of punicalagin, has good effects on preventing and treating myocardial fibrosis, and anti-inflammatory activity (Chen et al. 2022; Hering et al. 2021). Further mechanism studies found that urolithin A has a good effect on inhibiting NLRP3, mainly reflected in mitigating STING-NLRP3 axis-mediated inflammatory response by promoting Parkin-dependent mitophagy (Zhang et al. 2022), and activating the AMPK and autophagy to inhibit the endoplasmic reticulum stress-related TXNIP/NLRP3/IL-1β signaling pathway (Zhang et al. 2021). Since punicalagin and its metabolite urolithin A both have good myocardial protection and inhibition of NLRP3 activity, in the future experiment, in-depth study will be conducted at the cellular level to clarify the substances.

## Conclusions

The mechanism of punicalagin on VR after AMI is related to the NLRP3/Caspase-1 signaling pathway. This study is the first to demonstrate the anti-inflammatory effects of punicalagin in VR after AMI and the inflammatory mechanism of VR after AMI was further elucidated. The regulatory targets and mechanisms need to be further confirmed *in vitro*.
